# Cross-cultural adaptation, reliability, and preliminary construct validity of the Japanese version of the Parkinson’s disease pain classification system

**DOI:** 10.1016/j.prdoa.2026.100441

**Published:** 2026-04-08

**Authors:** Shuhei Ishida, Tomohiko Nishigami, Akira Mibu, Masahiro Manfuku, Hirofumi Yamashita, Yuta Tomooka, Norimasa Egusa, Shigeto Moriwaki, Kenichi Iwasa, Keiko Yamada, Satoshi Abe, Sokichi Maniwa

**Affiliations:** a Department of Rehabilitation, Shimane University Hospital, Izumo, Shimane, Japan; b Graduate School of Comprehensive Scientific Research, Prefectural University of Hiroshima, Mihara, Hiroshima, Japan; c Department of Physical Therapy, Faculty of Health and Welfare, Prefectural University of Hiroshima, Mihara, Hiroshima, Japan; d Department of Physical Therapy, Konan Women’s University, Kobe, Hyogo, Japan; e Department of Rehabilitation, Breast Care Sensyu Clinic, Kishiwada, Osaka, Japan; f Department of Rehabilitation, SKY Clinic, Ibaraki, Osaka, Japan; g Department of Rehabilitation, Fukuoka Orthopaedic Hospital, Fukuoka, Fukuoka, Japan; h Department of Neurology, Faculty of Medicine, Shimane University, Izumo, Shimane, Japan; i Pain Medicine, Juntendo University Graduate School of Medicine, Tokyo, Japan; j Department of Neurology, Shimane Prefectural Central Hospital, Izumo, Shimane, Japan; k Department of Rehabilitation, Faculty of Medicine, Shimane University, Izumo, Shimane, Japan

**Keywords:** Parkinson’sdisease, Chronicpain, Painphenotyping, Reliability, Cross-culturaladaptation

## Abstract

**Introduction:**

Pain is a burdensome non-motor symptom of Parkinson’s disease (PD). However, its mechanisms are complex, complicating pain phenotyping. The PD Pain Classification System (PD-PCS) classifies pain mechanisms and their relationship with PD pathology. We developed a Japanese PD-PCS and evaluated its reliability and preliminary construct validity.

**Methods:**

The PD-PCS was translated and adapted under developer supervision using an ISPOR-guided process. People with PD and chronic pain were recruited from two hospitals (n = 31). Intra- and inter-rater reliability were assessed with intraclass correlation coefficients (ICCs) for the PD-PCS pain score and κ for categorical classifications (95% CIs). The standard error of measurement (SEM) and the smallest detectable change (SDC) were calculated. Construct validity was examined using Spearman’s correlations between the PD-PCS pain score and the Brief Pain Inventory (BPI; average pain, interference).

**Results:**

Cognitive debriefing identified wording issues that were resolved through revisions, resulting in the final Japanese version. Participants (n = 31) had a mean age of 70.5 ± 8.3 years, and 16 (51.6%) were men. The intra-rater reliability was good (ICC[3,1] = 0.768, 95% CI, 0.575–0.880; SEM 10.4; SDC_individual 29.0), and the inter-rater reliability was excellent (ICC[2,1] = 0.917, 95% CI, 0.836–0.959; SEM 5.8; SDC_individual 16.0). The agreement between pain mechanisms and PD-related pain classification was substantial to perfect (κ = 0.755–1.000). The PD-PCS pain score correlated with BPI average pain (ρ = 0.744) and interference (ρ = 0.747).

**Conclusion:**

The Japanese PD-PCS demonstrated good to excellent reliability and preliminary construct validity, supporting its use in mechanism-informed pain classification in Japanese people with PD.

## Highlights

• Japanese PD-PCS developed using ISPOR-based cross-cultural adaptation.
• Cognitive debriefing finalized a clear and culturally appropriate version.
• Pain score showed good intra- and excellent inter-rater reliability.
• Scores correlated with pain burden and health-related quality of life.
• Measurement error suggests caution for individual longitudinal changes.

## 1 Introduction

The incidence of Parkinson’s disease (PD) is increasing worldwide owing to population growth, age, and lifestyle changes [1]. People with PD have motor symptoms such as akinesia, rigidity, tremors, and postural instability, as well as non-motor symptoms such as psychiatric symptoms and sleep disturbances, each characterized by a variety of symptoms. Pain is one of the most common problems among non-motor symptoms, with a reported prevalence of 67.6–84.8% [2], [3], [4]. The prevalence of pain increases as PD progresses [5], and pain is associated with quality of life (QOL) [6], sleep disturbance [7], and depression [7], [8]. Importantly, pain frequently occurs even in the early stages of the disease and has been reported to have a substantial impact on QOL, sometimes exceeding that of motor symptoms [3]. Therefore, the accurate assessment and management of pain are important clinical issues in people with PD.

Pain in people with PD is associated with a complex pathophysiology, and there is no clear consensus regarding its classification [9]. Previous pain classification methods have varied in their association with PD pathology and have mainly relied on symptom-based categories [10], [11], [12], [13]. The King’s Parkinson’s Disease Pain Scale (KPPS) is widely used to comprehensively assess different types of pain experienced by people with PD [13]. It evaluates multiple pain domains and provides a multidimensional assessment of pain severity. Higher KPPS total scores have been shown to correlate with poorer health-related QOL and other non-motor symptom burdens in people with PD [13]. However, the KPPS does not distinguish between the underlying pain mechanisms proposed by the International Association for the Study of Pain, namely, nociceptive, neuropathic, and nociplastic pain [14], limiting its ability to support mechanism-based pain phenotyping and targeted management strategies.

Nociplastic pain, defined as “pain that arises from altered nociception despite no clear evidence of actual or threatened tissue damage causing the activation of peripheral nociceptors or evidence for disease or lesion of the somatosensory system causing pain” [14], is clinically important because it can be associated with suboptimal response to peripherally targeted interventions and often requires individualized management [15]. To enable mechanism-based pain classification in PD, Mylius et al. developed the Parkinson’s Disease Pain Classification System (PD-PCS), which is an evaluator-based instrument administered via structured interviews and existing assessment tools [4]. The PD-PCS categorizes pain according to underlying mechanisms and has been reported to correlate with pain intensity, health-related QOL, motor and non-motor symptoms, and psychological factors in people with PD [4]. Accordingly, the PD-PCS may support mechanism-informed pain phenotyping and guide tailored clinical decision-making. For use in other languages and cultures, translation and cross-cultural adaptation as well as evaluation of measurement properties are essential. Although a German version of the PD-PCS exists [16], no Japanese version is currently available, despite the estimated 250,000 individuals with PD in Japan [17]. Cross-cultural adaptation is crucial not only to ensure linguistic and cultural appropriateness within a single country but also to facilitate consistent use across diverse languages and cultural contexts, thereby supporting reliable, valid, and comparable assessment of PD-related pain internationally. In addition to reliability, preliminary construct validity, such as the expected associations between the PD-PCS pain scores and external pain and health-related measures, should be examined to support interpretation of scores in clinical and research contexts. Establishing a Japanese version of the PD-PCS will allow clinicians to classify pain according to its underlying mechanism and implement mechanism-specific management strategies. Therefore, this study aimed to develop a Japanese version of the PD-PCS through a systematic translation and cross-cultural adaptation process and to evaluate its inter- and intra-rater reliability and preliminary construct validity.

## 2 Methods

### 2.1 Study design

The study was conducted in three phases. Phase 1 involved translation and cross-cultural adaptation. Phase 2 evaluated reliability, and Phase 3 assessed preliminary construct validity. This study was approved by the Medical Research Ethics Committee, Shimane University Faculty of Medicine (Nos. 8075 and 8337), and registered in the Japanese University Hospital Information Network Clinical Trials Registry (UMIN-CTR: UMIN000050190). Written informed consent was obtained from all participants prior to enrollment.

### 2.2 Phase 1: Translation and cross-cultural adaptation

**Fig. 1:**
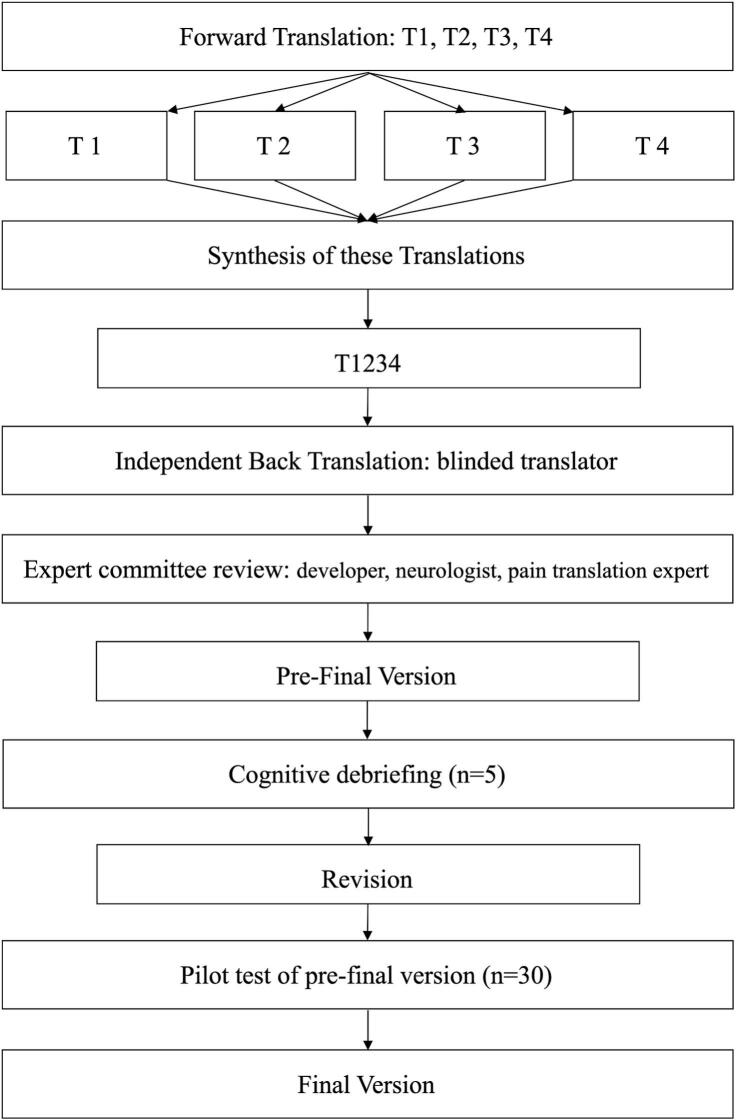
**Recommendations for translation and cross-cultural adaptation.** Flowchart of the translation and cross-cultural adaptation process for the Japanese version of the PD-PCS. Four bilingual translators independently conducted forward translations (T1–T4), followed by synthesis and independent back translation by a blinded translator. All versions were reviewed by an expert committee, including the original developer, a neurologist, and a pain translation expert, to ensure semantic, idiomatic, cultural, and conceptual equivalence and to develop a pre-final version. The pre-final version underwent cognitive debriefing (n = 5), revision, and pilot testing in an independent sample (n = 30), leading to the final version. *T1, physical therapist; T2–T3, pain researchers; T4, neurologist; PD-PCS, Parkinson’s Disease Pain Classification System.*

Before the study began, we contacted Dr. Mylius for permission to translate the instrument (PD-PCS). The PD-PCS was translated into Japanese following translation procedures in accordance with the recommendations of the International Society for Pharmacoeconomics and Outcomes Research (ISPOR) [18], with oversight from the original author. This study was conducted in the following order: forward translation, back translation, translation review, creation of the pre-final Japanese version, cognitive debriefing, pilot test, and creation of the Japanese version (Fig. 1).

Translation was conducted by a multidisciplinary team comprising a physical therapist (SI), two pain researchers (TN and AM), a neurologist (SA), the original developer of the PD-PCS (MV), a rehabilitation physician (SM), and a physician with expertise in pain management translation (KY). Four native Japanese translators (SI, TN, AM, and SA) independently performed forward translations. The translation team compared all forward versions, resolved discrepancies by consensus, and produced the first Japanese draft. The terminology was reviewed and supervised by a neurologist (SA). Next, an independent translator with relevant experience, who was blinded to the source version and had no involvement with the original instrument performed a back-translation based on the first Japanese draft. The team then reviewed the back-translation against the source version to ensure semantic, idiomatic, experiential, and conceptual equivalence and overall consistency, resulting in a pre-final Japanese version.

After the pre-final version was completed, we conducted cognitive debriefing and pilot testing to evaluate its clarity, comprehensibility, and cultural appropriateness. Cognitive debriefing (n = 5) and pilot testing (n = 30) were conducted on individuals with a neurologist-confirmed diagnosis of PD recruited from Shimane University Hospital. Eligibility largely followed the original PD-PCS study (including patients with or without pain and requiring an ON state during assessment) [4], except that the Mini-Mental State Examination (MMSE) cutoff was aligned to our protocol (MMSE < 24 excluded). Deep brain stimulation, or levodopa–carbidopa intestinal gel therapy, was also excluded. In contrast, Phases 2–3 included only participants with chronic pain lasting ≥ 3 months. Cognitive debriefing comprised a brief structured questionnaire (nine items; e.g., overall ease of understanding) (Supplementary Table 1) and individual interviews administered by a single evaluator (SI). Participants were asked to comment on any items rated “No” or “Neither,” and to describe difficulties in interpreting questions or response options. Interviews were conducted in private rooms, and participants’ comments and observed challenges were recorded. The findings were reviewed by three native Japanese speakers (SI, TN and AM), who refined problematic wording and revised the pre-final version accordingly. A pilot study was then conducted with an independent sample of 30 participants (separate from the reliability cohort and not included in Phases 2–3) to confirm feasibility and identify any remaining linguistic or cultural issues. Following these steps, the original developer (MV), neurologist (SA), and pain management translation expert (KY) reviewed the revised version and finalized the Japanese PD-PCS. The final Japanese version was provided in the Supplementary Material.

### 2.3 Phase 2: reliability study

#### 2.3.1 Participants and setting

Participants with neurologist-confirmed PD and chronic pain lasting ≥ 3 months were recruited from two hospitals (Shimane University Hospital and Shindenbara Seibo Hospital). All assessments in Phases 2–3 were conducted while participants were in the clinically defined ON state. We planned the sample size based on precision, rather than on hypothesis testing. Additionally, an exploratory assessment of the association between the PD-PCS pain score and external clinical measures was planned. Assuming an expected intraclass correlation coefficient (ICC[3,1]) of 0.80 with two ratings per participant (k = 2), a total sample of 30 yielded an expected 95% confidence interval half-width of approximately ± 0.14, which we deemed adequate to classify reliability as good vs. excellent. Note that under a hypothesis-testing framework (e.g., testing ICC ≤ 0.70 vs ≥ 0.85 at α = 0.05), n = 30 provides modest power; therefore, we prioritized precision-based planning to ensure interpretable confidence intervals. A sample size of approximately 30 was considered sufficient to detect large correlations (ρ = 0.5).

#### 2.3.2 Procedures

The PD-PCS is an evaluator-administered, structured classification system designed to assess pain in people with PD according to its relationship with PD pathology and the underlying pain mechanisms. In STEP 1, four screening questions were administered to evaluate whether the reported pain was potentially related to PD pathology. If at least one criterion was fulfilled, the pain was classified as PD-related. In STEP 2, pain was classified according to its predominant mechanism as nociceptive, neuropathic, or nociplastic based on existing diagnostic criteria. In addition, a PD-PCS pain score ranging from 0 to 90 was calculated by multiplying pain intensity (numerical rating scale [NRS]) by ratings of pain frequency and pain-related impact (three levels each), with higher scores indicating greater pain severity. Detailed item content and scoring procedures are provided in the original PD-PCS publication [4].

The measurement procedures followed the COSMIN guidelines [19]. Intra-rater reliability was assessed by having the same evaluator (Rater A) administer the Japanese PD-PCS at two visits (Visit 1 and Visit 2) approximately two weeks apart [20]. To confirm clinical stability over the retest interval, a physician rated the Clinical Global Impression (CGI) of change [21] at Visit 2. The CGI is a 7-point scale ranging from “Very much improved” to “Very much worse.” Consistent with previous studies [4], participants with CGI ratings from “Minimally improved” to “Minimally worse” were considered to have no clinically meaningful change and were eligible for re-evaluation.

Pain intensity during Visit 2 was also assessed by inquiring about the average pain experienced in the past 24 h. Inter-rater reliability was assessed at Visit 2, where a second evaluator (Rater B), who was blinded to Rater A’s scores, independently administered the PD-PCS during the same visit under comparable clinical conditions. Rater B was not present during Rater A’s interview and did not have access to its content. In the inter-rater reliability assessment, participants were evaluated sequentially by two raters on the same day with an interval of approximately 15 min between raters. Participants were informed that they did not need to reproduce their previous responses and were encouraged to answer independently for each rater. To minimize anchoring and recall effects, the raters followed standardized interview procedures and did not discuss participant responses or ratings during data collection. Four evaluators participated in the study, with two evaluators from each hospital. Across the two hospitals, two evaluators per site participated (three physical therapists and one occupational therapist), all with over five years of clinical experience in neurology. All assessments were performed independently with mutual blinding. Before data collection, the evaluators underwent a standardized 60-minute in-person oral training session. The training included the structure and content of the PD-PCS, methods for assessing its correspondence with pathological findings, guidance on items requiring verbal explanation during STEP 1, and standardized procedures for determining pain mechanisms. No formal calibration exercises or competency testing were conducted prior to data collection.

### 2.4 Phase 3: Preliminary construct validity study

Phase 3 was conducted in the same cohort as in Phase 2. External measures were used to test the hypotheses of preliminary construct validity. Sociodemographic and clinical data (age, sex, medications, and disease duration) were collected from the medical records. We calculated the levodopa equivalent daily dose (LEDD) for dopamine agents according to Schade et al. [22]. The motor severity of PD was assessed using the MDS-UPDRS part III and the Hoehn and Yahr (H&Y) stage. In the MDS-UPDRS part III, motor function is rated on a scale of 0–132 for various tasks, with higher scores indicating greater severity [23]. The H&Y stage was collected while evaluating the MDS-UPDRS score.

Pain intensity and interference were assessed using the Brief Pain Inventory (BPI) [24]. The BPI intensity is rated on a scale of 0–10, with 0 indicating none and 10 indicating the worst. Pain intensity is assessed as worst pain, least pain, average pain, and pain within 24 h. The BPI interference consists of seven items (General activity, Mood, Walking ability, Normal work, Relationships with other people, Sleep, Enjoyment of life) and is rated on a scale of 0–10.

The Widespread Pain Index (WPI) is a patient-reported measure that captures the extent of pain distribution across the body [25]. It records whether pain is present in 19 predefined anatomical regions (e.g., neck and lower back) and yields a total score ranging from 0 to 19. Higher WPI scores reflect a greater spatial spread of pain.

Health-related QOL was assessed using the EuroQol 5-dimension questionnaire (EQ-5D-5L), which is a patient-reported outcome [26]. The EQ-5D-5L has been reported to exhibit excellent psychometric properties in a wide range of populations, conditions, and settings [27], and is recommended for use in PD [28]. The EQ-5D-5L is a 5-point scale rating from no problems to severe problems for mobility, self-care, usual activities, pain/discomfort, and anxiety/depression. Scores are typically anchored at 1 (full health), with lower values indicating worse health (potentially below 0 depending on the set value).

### 2.5 Statistical analysis

#### 2.5.1 Phase 2: Reliability

Prior to statistical analysis, the data were checked for completeness. Descriptive statistics were calculated for all the assessment items. Intra-rater reliability was assessed using a two-way mixed-effects model with absolute agreement and a single-measure intraclass correlation coefficient (ICC[3,1]). Inter-rater reliability was assessed using a two-way random-effects model with absolute agreement and single-measure ICC[2,1]. Confidence intervals (CIs) of 95% were calculated for all estimates. Measurement error was quantified as the standard error of measurement (SEM) = SD × √(1 − ICC) and the smallest detectable change at the individual level (SDC_individual) = 1.96 × √2 × SEM. ICC values were interpreted as poor (ICC < 0.5), moderate (0.5 ≤ ICC ≤ 0.75), good (0.75 ≤ ICC ≤ 0.9), and excellent (ICC > 0.9). Categorical variables were assessed using Cohen’s kappa statistic (κ). Quadratically weighted κ was applied to ordinal variables (e.g., the number of criteria met in STEP 1), whereas unweighted Cohen’s κ was used for nominal classifications, including pain mechanisms and PD-relatedness. The magnitude of κ was interpreted as poor (≤ 0), slight (0.00–0.20), fair (0.21–0.40), moderate (0.41–0.60), substantial (0.61–0.80), or almost perfect (0.80–1.00), according to Landis and Koch [29]. Because pain intensity can fluctuate over short periods even when overall clinical status is stable, we conducted prespecified sensitivity analyses restricted to participants whose pain intensity was relatively stable between Visits 1 and 2. Specifically, we repeated intra- and inter-rater reliability analyses in two subsets defined by the change in NRS pain intensity between visits (ΔNRS ≤ 2 and ΔNRS ≤ 1). The same statistical approach was applied as in the primary reliability analyses (ICC models for continuous scores; weighted/unweighted κ for ordinal/nominal variables). These analyses were interpreted as robustness checks, acknowledging that reduced sample sizes widened the confidence intervals.

#### 2.5.2 Phase 3: Preliminary construct validity

For preliminary construct validity, Spearman’s rank correlation coefficients (ρ) between the PD-PCS pain score and external clinical measures were calculated. Spearman’s ρ values are reported with 95% CIs, and p-values are provided for descriptive purposes. The a priori hypothesis was that the PD-PCS pain score would correlate positively with pain intensity/interference and widespread pain, negatively with the EQ-5D-5L, and weakly with motor severity (MDS-UPDRS part III) and disease-related variables (disease duration and LEDD).

All statistical analyses were performed using R Studio, version 4.4.1 (R Foundation for Statistical Computing, Vienna, Austria).

## 3 Results

### 3.1 Phase 1: translation and cross-cultural adaptation

After resolving inconsistencies in translators’ expressions through forward translation, the versions were consolidated into a single version. When the consolidated version was back-translated into English, no major conceptual differences were found compared with the original version. These versions were reviewed to create a pre-final Japanese version.

During the cognitive debriefing, three participants indicated that it was difficult to determine whether the questions matched their symptoms. Additionally, two participants pointed out the complexity of this terminology. Discussions among the original author, neurologist, and translators addressed the clarification of terminology (e.g., peak-dose pain, non-motor OFF, painful dystonic spasms) and the expression of “intermediate” versus “moderate” in the Japanese version of the pain score. Clarifications of the necessary terms and revisions to the pain score expressions were made. In a pilot test involving 30 participants (mean age 72.7 ± 8.7 years, 60% female), of whom 28 reported pain, the instrument was feasible to administer and comprehensible. No further substantial modifications were required, and the final Japanese version of the PD-PCS was thus established.

### 3.2 Phase 2: reliability study

Two-hospital recruitment yielded 31 of the 38 eligible participants with complete data at both visits. Participants had a mean age of 70.5 ± 8.3 years, and 51.6% were men; mean disease duration was 7.8 ± 5.8 years, LEDD was 441.2 ± 220.7 mg/day, and MDS-UPDRS part III 32.4 ± 15.1. Most participants had H&Y stage II–III disease (Table 1). Among the 31 participants included in the analysis, 26 (83.9%) were classified as having PD-related pain and 5 (16.1%) as PD-unrelated pain. The classification of PD-PCS pain mechanisms was nociceptive pain 22 (71.0%), neuropathic pain 4 (12.9%), and nociplastic pain 5 (16.1%).

**Table 1:** Demographic and clinical characteristics of participants included in Phase 1 (translation and cross-cultural adaptation). (n = 31).

Variables		Mean ± SD, median (IQR), or n (%)
Age (years)		70.5 ± 8.3
Sex, n (%)	Male Female	16 (51.6) 15 (48.4)
PD duration (years)		7.8 ± 5.8
LEDD (mg/day)		441.2 ± 220.7
MDS-UPDRS part III		32.4 ± 15.1
Hoehn & Yahr stage, n (%)	I II III IV	2 (6.5) 17 (54.8) 10 (32.3) 2 (6.5)
EQ-5D-5L		0.666 ± 0.200
BPI-average		3.9 ± 1.9
BPI-worst		5.6 ± 2.1
BPI-interference		3.1 ± 2.5
Pain duration (months)		101.6 ± 150.4; 36.0 [16.5–120.0]
Pain distribution, n (%)	Neck Shoulders Upper arms Lower arms Upper back Low back Abdomen Lower legsFeet	3 (9.7) 1 (3.2) 1 (3.2) 1 (3.2) 4 (12.9) 11 (35.5) 2 (6.5) 6 (19.4) 2 (6.5)
WPI		2.9 ± 1.9
Number of criteria for PD-PCS STEP 1, n (%)	Zero criteria One criterion Two criteriaThree criteria	5 (16.1) 10 (32.3) 11 (35.5) 5 (16.1)
PD-related/unrelated, n (%)	PD-relatedPD-unrelated	26 (83.9) 5 (16.1)
Pain mechanism, n (%)	Nociceptive Neuropathic Nociplastic	22 (71.0) 4(12.9) 5(16.1)

Values are presented as mean ± SD or n (%). LEDD, Levodopa Equivalent Daily Dose; MDS−UPDRS, Movement Disorder Society Unified Parkinson's Disease Rating Scale; EQ-5D-5L, EuroQol 5-dimension questionnaire; BPI, Brief Pain Inventory; WPI, Widespread Pain Index; PD-PCS, Parkinson’s Disease Pain Classification System.

Intra-rater reliability was assessed after 10.8 ± 6.1 days. No participants was excluded based on the CGI (minimally improved, n = 4; no change, n = 26; minimally worse, n = 1) (Supplementary Table 2), in line with the prespecified clinical stability definition. Intra-rater agreement was substantial for the relationship with PD (κ = 0.795) and pain mechanisms (κ = 0.846), whereas agreement for the number of criteria for PD-PCS STEP 1 was substantial (κ = 0.687). The PD-PCS pain score showed good intra-rater reliability (ICC[3,1] = 0.768, 95% CI, 0.575–0.880), with an SEM of 10.4 and an SDC_individual of 29.0.

Inter-rater reliability at Visit 2 showed perfect agreement for the relationship with PD (κ = 1.000), substantial agreement for the number of criteria for PD-PCS STEP 1 (κ = 0.842) and pain mechanisms (κ = 0.755), and excellent reliability for the PD-PCS pain score (ICC[2,1] = 0.917, 95% CI, 0.836–0.959), with an SEM of 5.8 and SDC_individual of 16.0 (Table 2).

**Table 2:** Reliability of the Japanese version: intra-rater and inter-rater. (n = 31).

Domain/Variable	Statistics	Point estimate	95% CI	SEM	SDC_individual
**Intra-rater**					
Relationship with PD pathology	κ	0.795			
Number of criteria for PD-PCS STEP 1	κ	0.687			
PD-PCS pain score	ICC[3,1]	0.768	0.575–0.880	10.4	29.0
Pain mechanisms	κ	0.846			
**Inter-rater**					
Relationship with PD pathology	κ	1.000			
Number of criteria for PD-PCS STEP 1	κ	0.842			
PD-PCS pain score	ICC[2,1]	0.917	0.836–0.959	5.8	16.0
Pain mechanisms	κ	0.755			

Values are presented as point estimates with 95% confidence intervals (CIs). ICC models: intra-rater = two-way mixed-effects, absolute agreement, single measures ICC[3,1]; inter-rater = two-way random-effects, absolute agreement, single-measure ICC[2,1]. SEM = SD × √(1 − ICC); SDC_individual = 1.96 × √2 × SEM. Weighted κ (quadratic weights) was used for ordinal variables (e.g., number of criteria for STEP 1), whereas unweighted Cohen’s κ was used for nominal classifications (pain mechanisms; relationship with PD). *ICC, intraclass correlation coefficient; κ, Cohen’s kappa; CI, confidence interval; SEM, Standard Error of Measurement; SDC, Smallest Detectable Change*.

Sensitivity analyses restricted to participants with relatively stable pain intensity between visits (ΔNRS ≤ 2 and ≤ 1) showed similar or slightly higher reliability for the PD-PCS pain score and substantial agreement for pain mechanism classification, supporting the robustness of the primary findings (Supplementary Tables 3 and 4).

### 3.3 Phase 3: preliminary construct validity study

We hypothesized that the PD-PCS pain score would show moderate-to-strong positive correlations with pain intensity and interference (BPI), a positive correlation with widespread pain (WPI), and a moderate negative correlation with health-related QOL (EQ-5D-5L), whereas correlations with motor severity (MDS-UPDRS part III) and disease-related variables (disease duration and LEDD) would be weak. The Spearman’s rank correlations between the PD-PCS pain score and external clinical measures were presented in Table 3 to provide preliminary evidence of construct validity. The PD-PCS pain score showed moderate positive correlations with pain intensity and pain-related interference assessed by the BPI (BPI-average: ρ = 0.744, 95% CI, 0.529–0.869; BPI-worst: ρ = 0.715, 95% CI, 0.483–0.853; BPI-interference: ρ = 0.747, 95% CI, 0.529–0.869; all p < 0.01). A small-to-moderate positive correlation was observed with WPI (ρ = 0.397, 95% CI, 0.050–0.659; p = 0.03). The PD-PCS pain score was moderately negatively correlated with health-related QOL measured by the EQ-5D-5L (ρ =  − 0.496, 95% CI, −0.723–−0.172; p < 0.01). In contrast, correlations with motor severity and disease-related variables were weak and not statistically significant (MDS-UPDRS part III: ρ = 0.176, p = 0.34; disease duration: ρ = -0.302, p = 0.10; LEDD: ρ = 0.310, p = 0.09).

**Table 3:** Preliminary Construct validity: Spearman correlations between PD-PCS pain score and external measures. (n = 31).

Outcome	ρ	95% CI	p
BPI-average	0.744	0.529–0.869	< 0.01
BPI-worst	0.715	0.483–0.853	< 0.01
WPI	0.397	0.050–0.659	0.03
MDS-UPDRS part Ⅲ	0.176	−0.190–0.499	0.34
PD duration	−0.302	−0.593–0.059	0.10
LEDD	0.310	−0.050–0.599	0.09
BPI-interference	0.747	0.529–0.869	< 0.01
EQ-5D-5L	−0.496	−0.723–−0.172	< 0.01

Values are presented as point estimates with 95% confidence intervals (CIs). *BPI, Brief Pain Inventory; WPI, Widespread Pain Index; MDS-UPDRS, Movement Disorder Society Unified Parkinson's Disease Rating Scale; LEDD, Levodopa Equivalent Daily Dose; EQ-5D-5L, EuroQol 5-dimension questionnaire*.

## 4 Discussion

This study developed a Japanese version of the PD-PCS through systematic translation and cross-cultural adaptation, and evaluated its reliability and preliminary construct validity in people with PD and chronic pain. Inter- and intra-rater reliabilities for the PD-PCS pain score ranged from good to excellent, and the agreement for classifications related to pain mechanisms and PD pathology ranged from substantial to almost perfect.

In the cognitive debriefing, several participants reported difficulty judging whether some questions matched their symptoms, whereas others noted that certain terms were complex. To address these issues, we refined potentially ambiguous terminology (e.g., peak-dose pain, non-motor OFF, painful dystonic spasms) and clarified the distinction between “intermediate” and “moderate” in the Japanese pain-score descriptors through iterative discussions among the original author, the neurologist, and the translators. After these refinements, a pilot test showed that the Japanese PD-PCS was feasible to administer and was generally comprehensible. No further substantial modifications were required, and the Japanese version of the PD-PCS was finalized.

The reliability indices observed in this study were comparable to those reported previously [4]. The PD-PCS pain score demonstrated good intra-rater reliability (ICC[3,1] = 0.768) and excellent inter-rater reliability (ICC[2,1] = 0.917), meeting commonly accepted standards [30]. Agreement for classifications of pain mechanisms (κ = 0.755–0.846) and PD-relatedness (κ = 0.795–1.000) ranged from substantial to almost perfect [29], whereas agreement for the number of criteria for PD-PCS STEP 1 was substantial in the intra-rater analysis (κ = 0.687). Notably, the absolute measurement error was nontrivial (SEM/SDC_individual: 10.4/29.0 intra-rater and 5.8/16.0 inter-rater), indicating that relatively large score changes were required to exceed the measurement error at the individual level. Given the PD-PCS pain score range (0–90), the SDC_individual values observed here (29.0 for intra-rater and 16.0 for inter-rater) corresponded to approximately one-third and one-sixth of the total scale range, respectively, indicating that relatively large within-person changes were required to exceed the measurement error. These measurement error indices have practical implications for how the PD-PCS pain scores. At the sample level, the score appears to be well-suited for research applications, such as between-group comparisons, correlational analyses, and stratification/phenotyping. In contrast, in longitudinal clinical follow-ups, small-to-moderate within-person changes should not be interpreted as definitive improvement or deterioration unless they clearly exceed the measurement error. Accordingly, whenever feasible, follow-up assessments should be conducted by the same evaluator using standardized procedures, and clinical interpretation should be supported by repeated measurements (or averaging across assessments), triangulation with complementary pain outcomes (e.g., NRS and BPI), and the global impression of change. Future studies should evaluate the responsiveness and clinically meaningful change thresholds to support individual-level monitoring of treatment effects.

Although this was not designed as a comprehensive validation study, the observed correlation pattern provided preliminary support for the construct validity of the PD-PCS pain score. Importantly, the PD-PCS pain score showed weak and non-significant associations with motor severity and disease-related variables (MDS-UPDRS part III, disease duration, and LEDD), supporting discriminant validity and suggesting that the score reflects the pain burden rather than overall disease severity or progression. The PD-PCS pain score also correlated with BPI and inversely with EQ-5D-5L, consistent with convergent validity. However, convergent correlations with BPI should be interpreted cautiously because pain intensity is embedded in the PD-PCS score, resulting in partial shared variance. The relatively low WPI scores suggest that widespread pain among participants was limited. This restricted range may have weakened its correlation with the PD-PCS pain score. Furthermore, the PD-PCS pain score and WPI assess different aspects of pain, which may explain the moderate correlation observed. These findings should be interpreted as preliminary, given the modest sample size. Future studies should assess incremental validity by testing whether associations with broader outcomes (e.g., EQ-5D-5L or WPI) persist after accounting for pain intensity (e.g., BPI-average).

This study has some limitations that should be acknowledged. First, the number of evaluators involved in the inter-rater reliability assessment was limited, and all were experienced therapists. While this reduced variability due to rater inexperience may restrict generalizability to less experienced raters, future studies should include a larger and more heterogeneous group of evaluators. In addition, raters received only brief training prior to data collection, and no formal calibration exercises or competency testing were conducted. Although this may limit methodological rigor, the acceptable reliability observed in this study suggests that the PD-PCS may be implementable in routine clinical practice with limited training. Second, inter-rater assessments were performed sequentially during the same visit. Although the raters were mutually blinded and followed standardized procedures, this design may have introduced anchoring or recall effects (e.g., participants repeating similar answers), potentially inflating agreement compared with fully independent assessments conducted on separate occasions. The interview order was not randomized, and the interval between interviews was not pre-specified, which may have allowed for carryover effects. Future studies should randomize the interview order, prespecify a washout interval, and explicitly instruct participants that they are not required to provide the same responses as in the previous interview. Third, stability during the retest interval was determined primarily using the CGI, which reflects the overall clinical status rather than pain-specific stability. Moreover, pain symptoms may fluctuate over short periods even in clinically stable patients; thus, part of the observed measurement error may reflect true changes in pain rather than rater inconsistency alone. Finally, although we provided preliminary evidence supporting construct validity via hypothesis-consistent correlations, other measurement properties remain to be established, including known-group validity, responsiveness, and clinically meaningful change thresholds.

## 5 Conclusions

We developed a Japanese PD-PCS with good-to-excellent reliability and preliminary construct validity. Our findings support its use in mechanism-informed pain classification in Japanese people with PD. Further studies should confirm its validity, evaluate responsiveness, and establish clinically meaningful changes.

## 6 Declaration of generative AI and AI-assisted technologies in the manuscript preparation process

During the preparation of this work, the authors used ChatGPT (OpenAI) to assist with language refinement and clarity only. The tool was not used for data analysis, interpretation, or generation of scientific content. The authors reviewed and edited the output as needed and take full responsibility for the content of the published article.

## 7 Data availability statement

The data that support the findings of this study are not publicly available due to ethical restrictions but are available from the corresponding author upon reasonable request.

## 8 Funding sources

This research did not receive any specific grant from funding agencies in the public, commercial, or not-for-profit sectors.

## CRediT authorship contribution statement

**Shuhei Ishida:** Writing – review & editing, Writing – original draft, Visualization, Validation, Resources, Methodology, Investigation, Formal analysis, Data curation, Conceptualization. **Tomohiko Nishigami:** Writing – review & editing, Writing – original draft, Visualization, Validation, Supervision, Project administration, Methodology, Formal analysis, Conceptualization. **Akira Mibu:** Writing – review & editing, Visualization, Validation, Methodology, Conceptualization. **Masahiro Manfuku:** Writing – review & editing, Visualization, Methodology, Conceptualization. **Hirofumi Yamashita:** Writing – review & editing, Visualization, Methodology, Conceptualization. **Yuta Tomooka:** Writing – review & editing, Visualization, Methodology, Conceptualization. **Norimasa Egusa:** Writing – review & editing, Resources, Methodology, Investigation. **Shigeto Moriwaki:** Writing – review & editing, Resources, Methodology, Investigation, Data curation. **Kenichi Iwasa:** Writing – review & editing, Supervision, Resources. **Keiko Yamada:** Writing – review & editing, Methodology. **Satoshi Abe:** Writing – review & editing, Resources, Conceptualization. **Sokichi Maniwa:** Writing – review & editing, Resources, Project administration, Data curation, Conceptualization.

## Declaration of competing interest

The authors declare that they have no known competing financial interests or personal relationships that could have appeared to influence the work reported in this paper.
