# Supplementary material for: Cross-cultural adaptation, reliability, and preliminary construct validity of the Japanese version of the Parkinson’s disease pain classification system
Source: Clin Park Relat Disord. 2026 Apr 8;14:100441. doi: 10.1016/j.prdoa.2026.100441 (PMC13092571; doi:10.1016/j.prdoa.2026.100441)
Supplement: Supplementary Data 1 [file mmc1.docx]

**The Parkinson’s Disease Pain Classification System (PCS) Questionnaire**

名前　　　　　　　　　　　　　生年月日　　　　　　　　　　　　　　　日付

罹患期間　　　　　　　　　　　症状優位側

パーキンソン病薬

患者は慢性的な疼痛を抱えています．疼痛の種類ごとに，その期間や，症状優位側，治療法を考慮し，情報を受け取ってください．それぞれの疼痛について，その場所と期間を以下に記載してください．

|  | 疼痛1 | 疼痛2 | 疼痛3 |
| --- | --- | --- | --- |
| 場所 |  |  |  |
| 期間 |  |  |  |

**Step１：4つの質問は，患者の疼痛がパーキンソン病と関連しているか確かめることを目的としています（Step2に進むためには，最低でも1つの質問にはいと答える必要があります．1つもない場合，パーキンソン病と関連が無く，Step3に進みます）．**

|  | 疼痛1 | 疼痛2 | 疼痛3 |
| --- | --- | --- | --- |
| パーキンソン病の症状が出てから，痛みが始まった，または，よりひどくなりましたか？ |  |  |  |
| 強剛，振戦，動作の緩慢さがより強くなると痛みが悪化しますか？ |  |  |  |
| 痛みは，過剰かつ異常な運動（ジスキネジア）と関係していますか？ |  |  |  |
| パーキンソン病薬を飲むと，痛みはいくらか改善しますか？ |  |  |  |

**Step２：パーキンソン病に関連した疼痛を，主要な３つのメカニズムの１つに分類してください．**

**神経障害性疼痛**は，体性感覚神経系の病変または疾患によって引き起こされる疼痛と定義されます．神経障害性疼痛質問票（DN4）が陽性（DN4≧4）の場合，神経障害性疼痛とみなします．中枢神経障害性疼痛は，その局在（末梢神経，神経根性，遠位部の対称性 vs びまん性）により末梢性神経障害性疼痛と区別できます．

神経障害性疼痛質問票（Couleur Neuropathique-4 questionnaire (DN-4)）：痛みにいずれかの特徴がありますか．1．焼けるような痛み；2．冷たく感じる痛み；3．電気が走る様な痛み；その同じ部位の痛みは次のいずれの症状を伴いますか．4．ピリピリ感じる；5．針に刺さるように感じる；6．にぶい又はしびれているように感じる；7．かゆく感じる；痛みを感じる部位の触診を受けた際，下記の特徴があるか．8．触れられることに対する知覚低下；9．チクチク刺すような痛みに対する知覚低下；痛い部位においてその痛みは，下記によって引き起こされたり増したりするか．10．軽くなでる

|  | 疼痛1 | 疼痛2 | 疼痛3 |
| --- | --- | --- | --- |
| 中枢性 |  |  |  |
| 末梢性 |  |  |  |
| スコア：　強度 × 頻度 ×　影響度 |  |  |  |

**侵害受容性疼痛**は神経組織以外の生体組織に対する実質的ないしは潜在的な傷害によって，侵害受容器が興奮して起こります．患者は，筋や腱， 筋膜の触診で疼痛を感じたり，疼痛を伴う強剛がみられますか？これらは，運動症状の変動による筋骨格系の疼痛として，オフ期の疼痛（early morning pain，wearing-off pain，beginning-of-dose pain，end-of-dose pain），多くの有痛性ジストニア（early morning dystonia，off-period dystonia，beginning-of-dose dystonia，end-of-dose dystonia），薬物血中濃度ピーク時の疼痛（peak-of-dose pain）を含みます．限局性あるいは局所性の疼痛症候群，筋筋膜性疼痛症候群，コートハンガー頭痛（低血圧に伴う後頚部・肩甲部痛）もここに含まれます．

|  | 疼痛1 | 疼痛2 | 疼痛3 |
| --- | --- | --- | --- |
| 局所性の疼痛 |  |  |  |
| 筋筋膜性疼痛症候群 |  |  |  |
| コートハンガー頭痛 |  |  |  |
| スコア：　強度 × 頻度 ×　影響度 |  |  |  |

疼痛が神経障害性疼痛でも侵害受容性疼痛でもない場合，**痛覚変調性疼痛**とみなします．臨床の場において， これらの疼痛は，ドパミン過剰/低下による変動性があり，非運動性の精神神経症状が臨床像として優勢です．疼痛が主訴ではなく，より複雑な臨床像の一部であることも多くあります．患者は，発汗による顔面の紅潮や不快感，身の置き所のなさ，焦燥感，移動性の疼痛，腹部の深部痛や顔面痛，また，場合によっては，局在化しておらず，急速に疼痛部位が移動することもあります．多くの場合，痛覚変調性疼痛を有するパーキンソン病患者は，ドパミンアゴニスト離脱症候群やドパミン調節障害，その他の精神神経症状に分類することができます．また，神経障害性疼痛の要素が優位でない場合，下肢静止不能（Leg motor restlessness）と非運動症状のオフをここに分類します．

|  | 疼痛1 | 疼痛2 | 疼痛3 |
| --- | --- | --- | --- |
| 非運動症状のオフ |  |  |  |
| 下肢静止不能（Leg motor restlessness） |  |  |  |
| ドパミンアゴニスト離脱症候群 |  |  |  |
| その他 |  |  |  |
| スコア：　強度 × 頻度 ×　影響度 |  |  |  |

**Step３：パーキンソン病に関連しない疼痛**

|  | 疼痛1 | 疼痛2 | 疼痛3 |
| --- | --- | --- | --- |
| 局在性 |  |  |  |
| メカニズム |  |  |  |
| スコア：　強度 × 頻度 ×　影響度 |  |  |  |

**疼痛のサブタイプのスコアリング**

疼痛の強さ（0=「痛くない」，10=「想像できる最大の痛み」とし，自身の疼痛の強さを選ぶ），頻度（1：まれ， 2：中間， 3：頻繁），日常生活への影響度（1：低， 2：中， 3：高）により各タイプ別にスコア化します．それぞれの疼痛の記述の合計得点は，強度，頻度，影響度の積算で得られ，それぞれの疼痛が0〜90の得点となります．
