# Supplementary material for: Cross-cultural adaptation, reliability, and preliminary construct validity of the Japanese version of the Parkinson’s disease pain classification system
Source: Clin Park Relat Disord. 2026 Apr 8;14:100441. doi: 10.1016/j.prdoa.2026.100441 (PMC13092571; doi:10.1016/j.prdoa.2026.100441)
Supplement: Supplementary Data 2 [file mmc2.docx]

**Table S1 Cognitive debriefing questionnaire items used in Phase 1 (translation and cross-cultural adaptation).**

| Items |
| --- |
| Was it easy to understand overall? |
| Was the time required for the response appropriate? |
| Was the number of questions appropriate? |
| Would you mind answering this questionnaire again? |
| Was the description easy to understand? |
| Did you understand the questionnaire easily? |
| Did you understand the meaning of the questions? |
| Was the question difficult to answer? |
| Were the answer options clear and responsive to questions? |

*Items were rated using a three-point response format (Yes/No/Neither).*

**Table S2 PD-PCS classifications and scores across two assessment occasions in Phase 2 (reliability study). (n = 31)**

| Domain/Variable | Visit 1 | Visit 2  same evaluator | Visit 2  second evaluator |
| --- | --- | --- | --- |
| PD-PCS |  |  |  |
| PD-related/PD-unrelated | 26 (83.9) / 5 (16.1) | 24 (77.4) / 7 (22.6) | 24 (77.4) / 7 (22.6) |
| Nociceptive pain | 22 (71.0) | 24 (77.4) | 23 (74.2) |
| Neuropathic pain | 4 (12.9) | 4 (12.9) | 6 (19.4) |
| Nociplastic pain | 5 (16.1) | 3 (9.7) | 2 (6.5) |
| PD-PCS pain score | 24.4 ± 21.7 | 27.6 ± 20.0 | 29.4 ± 22.9 |
| NRS | 3.9 ± 1.9 | 5.4 ± 1.7 | 5.7 ± 2.3 |
| Change scores |  |  |  |
| CGI |  | 3.9 ± 0.4 |  |
| Minimally improved |  | 4 (12.9) |  |
| No change |  | 26 (83.9) |  |
| Minimally worse |  | 1 (3.2) |  |
| Interval between assessments (days) |  | 10.8 ± 6.1 |  |

Values are presented as mean ± SD or n (%). *PD-PCS, Parkinson’s Disease Pain Classification System; NRS, Numerical Rating Scale; CGI, Clinical Global Impression scale*

**Table S3 Reliability of the Japanese version for participants with ΔNRS ≤ 2: Intra-rater and inter-rater. (n = 23)**

| Domain/Variable | Statistics | Point estimate | 95% CI | SEM | SDC_individual |
| --- | --- | --- | --- | --- | --- |
| **Intra-rater** |  |  |  |  |  |
| Relationship with PD pathology | κ | 0.701 |  |  |  |
| Number of criteria for PD-PCS STEP 1 | κ | 0.622 |  |  |  |
| PD-PCS pain score | ICC [3,1] | 0.821 | 0.628–0.919 | 8.9 | 24.7 |
| Pain mechanisms | κ | 0.902 |  |  |  |
| **Inter-rater** |  |  |  |  |  |
| Relationship with PD pathology | κ | 1.000 |  |  |  |
| Number of criteria for PD-PCS STEP 1 | κ | 0.874 |  |  |  |
| PD-PCS pain score | ICC [2,1] | 0.926 | 0.833–0.968 | 6.3 | 17.4 |
| Pain mechanisms | κ | 0.701 |  |  |  |

Values are presented as point estimates with 95% confidence intervals (CIs). ICC models: intra-rater = two-way mixed-effects, absolute agreement, single measures ICC [3,1]; inter-rater = two-way random-effects, absolute agreement, single- measure ICC [2,1]. SEM = SD × √(1 − ICC); SDC_individual = 1.96 × √2 × SEM. Weighted κ (quadratic weights) was used for ordinal variables (e.g., number of criteria for STEP 1), whereas unweighted Cohen’s κ was used for nominal classifications (pain mechanisms; relationship with PD). *ICC, intraclass correlation coefficient; κ, Cohen’s kappa; CI, confidence interval; SEM, Standard Error of Measurement; SDC, Smallest Detectable Change*

**Table S4 Reliability of the Japanese version for participants with ΔNRS ≤ 1: Intra-rater and inter-rater. (n = 18)**

| Domain/Variable | Statistics | Point estimate | 95% CI | SEM | SDC_individual |
| --- | --- | --- | --- | --- | --- |
| **Intra-rater** |  |  |  |  |  |
| Relationship with PD pathology | κ | 0.824 |  |  |  |
| Number of criteria for PD-PCS STEP 1 | κ | 0.849 |  |  |  |
| PD-PCS pain score | ICC [3,1] | 0.807 | 0.552–0.923 | 8.8 | 24.5 |
| Pain mechanisms | κ | 0.863 |  |  |  |
| **Inter-rater** |  |  |  |  |  |
| Relationship with PD pathology | κ | 1.000 |  |  |  |
| Number of criteria for PD-PCS STEP 1 | κ | 0.886 |  |  |  |
| PD-PCS pain score | ICC [2,1] | 0.910 | 0.775–0.965 | 6.5 | 18.0 |
| Pain mechanisms | κ | 0.743 |  |  |  |

Values are presented as point estimates with 95% confidence intervals (CIs). ICC models: intra-rater = two-way mixed-effects, absolute agreement, single measures ICC [3,1]; inter-rater = two-way random-effects, absolute agreement, single- measure ICC [2,1]. SEM = SD × √(1 − ICC); SDC_individual = 1.96 × √2 × SEM. Weighted κ (quadratic weights) was used for ordinal variables (e.g., number of criteria for STEP 1), whereas unweighted Cohen’s κ was used for nominal classifications (pain mechanisms; relationship with PD). *ICC, intraclass correlation coefficient; κ, Cohen’s kappa; CI, confidence interval; SEM, Standard Error of Measurement; SDC, Smallest Detectable Change*
